# Interactions of Potassium Fertilization and Straw Return in Modulating Maize Yield and Lodging Resistance

**DOI:** 10.3390/plants14233665

**Published:** 2025-12-02

**Authors:** Xiaowen Wang, Jia Liu, Shuang Liu, Yao Zhao, Hong Ren, Yan Gu

**Affiliations:** 1 College of Agronomy, Jilin Agricultural University, Changchun 130118, China; wangxw@mails.jlau.edu.cn (X.W.); liujia_jlau@163.com (J.L.); 1042642402@139.com (S.L.); 2 Institute of Crop Sciences, Chinese Academy of Agricultural Sciences, Beijing 100081, China; 82101225054@caas.cn

**Keywords:** maize, straw return, potassium fertilizer, lodging resistance index, yield

## Abstract

Maize lodging is a major factor limiting maize grain yield. Potassium (K) fertilization is known to reduce lodging, but the potential impact of straw return on lodging resistance remains unclear. A two-year field experiment was conducted with five K levels (0, 30, 60, 90, and 120 kg ha^−1^) under straw return (S1) and no straw return (S0). Maize yield, stem lodging resistance index (SLRI), crushing strength (CS), stem morphological and physicochemical characteristics, and soil nutrient levels were measured. Compared to S0, increased K application with S1 significantly enhanced the SLRI (16.0%) and CS (19.8%) across two years, which was due to the improvement of stem morphological (internode dry weight, length, and plumpness) and physiological characteristics (soluble sugar, cellulose, lignin, phenylalanine ammonia-lyase (PAL), tyrosine ammonia-lyase (TAL), and cinnamyl alcohol dehydrogenase (CAD)), especially the third internode. The highest SLRI and CS of each internode of the two straw treatments were obtained in K120, while no significant difference between K90 and K120 was observed for these indicators under the same straw treatment. Grain yield and soil available K content of S1 were higher by an average of 5.0% and 18.0% than S0, respectively. Compared to K0, K120 increased the yield and soil available K content by 17.3% and 18.8%, but there was no significant difference with K90. As a result, S0 and S1 both achieved a soil K balance when the surplus rate was close to zero at a K input of 90 kg ha^−1^. Fitting analysis indicated that, compared to S0, the K application rate of S1 was reduced by an average of 11.8% while maintaining a K surplus rate of 0, which means S1 could enhance soil potassium cycling and supply capacity but also reduce fertilizer input. In conclusion, straw return combined with K fertilizer (e.g., 90 kg ha^−1^) is an effective strategy to enhance lodging resistance and maintain maize yield by improving stem morphological and physicochemical characteristics.

## 1. Introduction

Maize (*Zea mays* L.) is a crucial food crop in China, functioning both as a staple food and a significant feed source [1,2]. Therefore, improving maize yield is essential for achieving food security [1]. However, in recent years, various adversities have arisen, and lodging has become the main obstacle limiting the increase in maize yield. Lodging disrupts the normal canopy structure of the plants, resulting in reduced photosynthetic activity, reduced dry matter synthesis and accumulation, and thus reduced grain yield [3]. Lodging can be classified into two types: one is root lodging, which refers to the tilting of the plant when the root system is under stress, or the entire plant lodging due to damage to the stem; stem lodging refers to lodging caused by the breaking of the stem. Compared to root lodging, stem lodging results in more severe grain loss. In addition, lodging may limit mechanical harvest, increase harvesting costs, and lead to a decrease in grain quality [4,5].

Studies have demonstrated that crop stem morphological characteristics (plant height and stem diameter), anatomical characteristics (vascular bundles and thick-walled tissues), and biochemical indicators (cellulose and lignin) are all intrinsic factors that affect crop lodging [6,7,8,9]. For maize plants, compared to the shorter basal internodes and thicker stems, plants with longer basal internodes and thinner stems have a higher center of gravity height and ear position and are more prone to lodging [9]. It was found that the stem mechanical strength, such as the puncture strength, crush strength, and bending strength, was an important indicator to assess the stem lodging resistance, and they were significantly negatively correlated with the lodging rate [10]. Therefore, increasing the stem strength at the base internodes can also reduce the risk of maize lodging [11]. In addition, cellulose, lignin, hemicellulose, and other structural carbohydrates in the stem are the material basis for the formation of mechanical cells and mechanical tissues, and their content determines the stem strength of maize [12]. These indicators are often closely related to farming methods [13]. Therefore, studying the lodging resistance of maize and improving its lodging resistance by regulating the cultivation measures is significant in reducing or preventing lodging occurrence.

In addition to genetic causes, stem lodging is also regulated by natural environment and cultivation measures, but natural factors, including climatic factors and ecological factors, are largely beyond human control [14,15]; therefore, the rational application of cultivation measures is particularly important for improving crop lodging ability. In agronomic measures, planting density, fertilization, irrigation, and crop management measures significantly affect lodging sensitivity. For example, excessive application of nitrogen fertilizer will weaken the cell wall structure and make plants more prone to lodging. Increasing density and irrigation will affect the resistance of crops, while the application of an appropriate amount of potassium and silicon fertilizer can increase stem strength and reduce the risk of lodging [16,17].

Potassium (K) is considered to be one of the important factors in increasing crop lodging resistance [18]. Potassium application promotes its distribution to the stem by altering internode traits, thereby enhancing stem lodging resistance of maize [19]. However, a widespread K deficiency occurred due to prolonged and disproportionate fertilization compared to nitrogen and phosphorus [20]. At present, K deficiency has become a worldwide problem [21,22,23]. In China, potassium resources are scarce but in great demand. China holds only 8.4% of the global K reserve but accounts for 24.4% of the total consumption [24]. Most of the potash fertilizers rely on imports. Since 2018, China’s dependence on foreign sources of potash fertilizer has been approximately 60% [25], and the increasing fertilizer prices over the years have undoubtedly increased the cost of agricultural production [26]. Soil potassium deficiency is a problem that needs to be alleviated urgently, but long-term input of potassium fertilizer to improve soil potassium is not a sustainable measure [27]. Considering the current agricultural soil production environment in China, it is crucial to focus on the scientific application of chemical fertilizers and explore measures to optimize the input of chemical fertilizers into soil [28].

Straw, as an important organic fertilizer resource, contains a large number of essential elements, such as nitrogen (N), phosphorus (P), and potassium (K) in the concentration ranges of 0.65–1.82%, 0.08–0.196%, and 1.02–1.94%, respectively [29]. With the development of agricultural mechanization and the implementation of the Chinese government’s policy on the return of crop residues and organic fertilizers, more crop residues are being returned to the soil, thereby increasing soil nutrients [30]. The recycling and utilization of straw with high potassium content provides great potential for reducing unbalanced input on chemical fertilizers. Zhu et al. [31] found that straw returned to soil can significantly increase nitrogen and potassium content in soil, thereby reducing the input of potassium fertilizer [32]. Moreover, straw return can effectively enhance soil fertility and crop yield [33], and it seems to be related to improving stem lodging resistance. When crops encounter adverse weather such as rainstorms and strong winds, the infiltration of rainwater makes the soil soft, the anchoring ability of roots is weakened, and crops are more prone to lodging [34]. The recycling and utilization of straw could optimize soil structure, improve soil fertility, soil aggregate stability, and the diversity of fungi, and enhance the nutrient absorption capacity of crop roots [35,36]. The study of lodging resistance is closely related to potassium, and straw is considered an important source for alleviating soil potassium deficiency and enriching soil potassium reservoirs. Therefore, additional studies are required to assess the response of lodging resistance to straw return combined with potassium fertilizer application, as well as the potential of straw return to reduce potassium fertilizer application.

In summary, potassium application and straw return both could have a positive effect on soil nutrients and grain yield, and potassium has a significant effect on improving crop lodging resistance [31,32,33]. Until now, most studies have examined the effects of straw return and chemical fertilizers on soil nutrients and yield, but the mechanism of the impact of their co-application on maize lodging has not yet been well documented. The aims of the current study were to (i) investigate differences in the effects of straw return and no straw return on the stem structure, physiological characteristics, and lodging resistance of maize after increasing the potassium application rate; and (ii) to clarify the optimal application rate of potassium fertilizer for achieving relatively high yield while maintaining soil potassium balance under straw return conditions.

## 2. Results

### 2.1. Grain Yield

**Figure 1:**
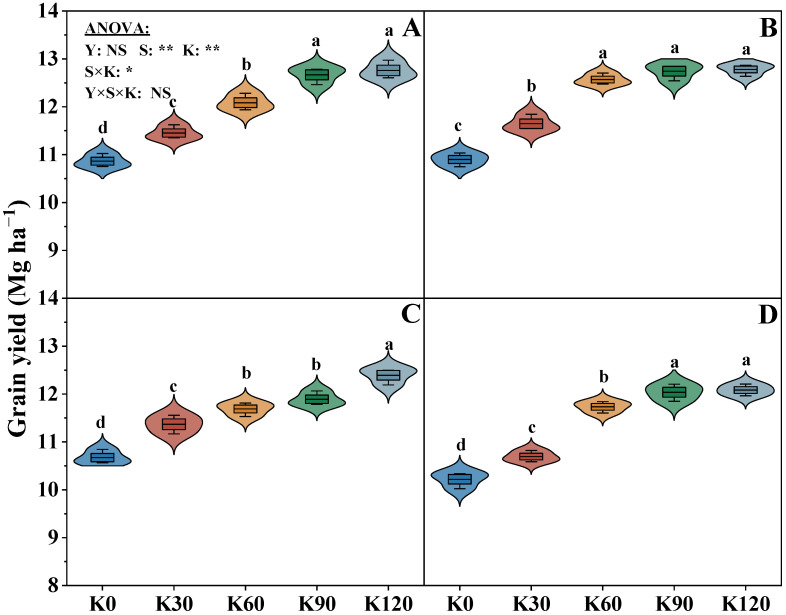
Effects of straw returning and potassium fertilizer on maize yield. (**A**–**D**) represent the maize yield, where (**A**,**B**) represent the maize yield of S1 treatment in 2018 and 2019, and (**C**,**D**) represent the maize yield of S0 treatment in 2018 and 2019. Y, S, and K represent year, straw treatment, and potassium fertilizer treatment, respectively. Values within a column followed by different lowercase letters indicate significant differences within treatments (*p* < 0.05). The error bar indicates the standard deviation (S.D., n = 3). * and ** indicate differences significant at the 0.05 and 0.01 levels, respectively. NS, no significance.

The years (Y), straw (S), and potassium fertilizer (K) treatments and their interactions (S × K, Y × S × K) significantly affected the yield (Y has no effect on yield) (Figure 1). The grain yield significantly increased with the increasing K input, which also confirms the significant impact of potassium on yield. The maize yield of the two straw treatments was the highest in the K120, while no significant difference between K90 and K120 was observed. Under the same potassium application rate, the maize yield of the S1 treatment was significantly higher by an average of 5.0% than that of the S0 treatment.

### 2.2. Stem Lodging Resistance Index (SLRI) and Crushing Strength (CS)

**Figure 2:**
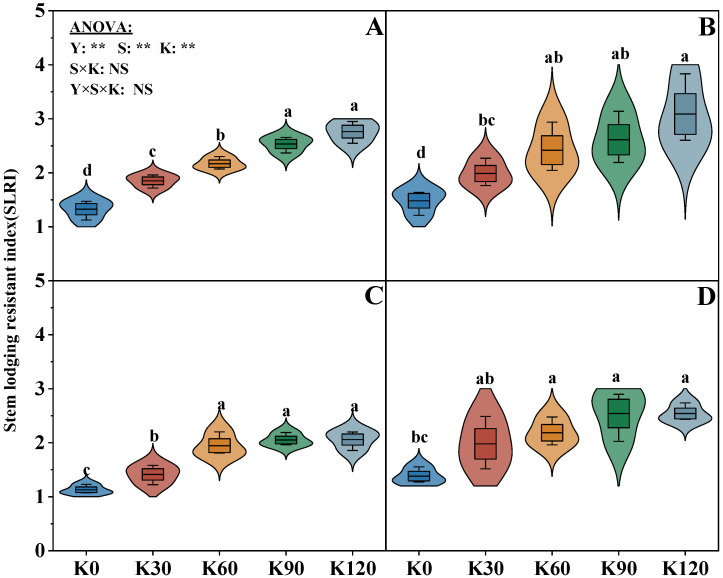
Effects of straw returning and potassium fertilizer on the stem lodging resistance index. (**A**–**D**) represent the lodging resistance index, where (**A**,**B**) represent the lodging resistance index of S1 treatment in 2018 and 2019, and (**C**,**D**) represent the lodging resistance index of S0 treatment in 2018 and 2019. Y, S, and K represent year, straw treatment, and potassium fertilizer treatment, respectively. Values within a column followed by different lowercase letters indicate significant differences within treatments (*p* < 0.05). The error bar indicates the standard deviation (S.D., n = 3). * and ** indicate differences significant at the 0.05 and 0.01 levels, respectively. NS, no significance.

**Figure 3:**
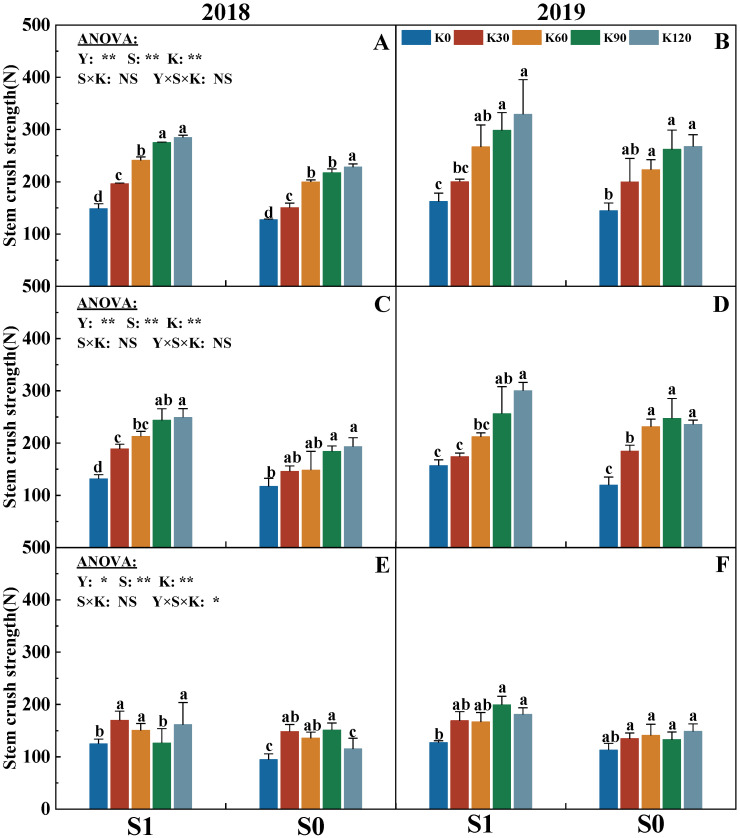
Effects of straw return and potassium fertilizer on the crush strength of the third (**A**,**B**), fourth (**C**,**D**), and fifth (**E**,**F**) internodes. Y, S, and K represent year, straw treatment, and potassium fertilizer treatment, respectively. Values within a column followed by different lowercase letters indicate significant differences within treatments (*p* < 0.05). The error bar indicates the standard deviation (S.D., n = 3). * and ** indicate differences significant at the 0.05 and 0.01 levels, respectively. NS, no significance.

The SLRI and CS were significantly affected by the factors of the years (Y), straw (S), and potassium fertilizer (K), but not affected by their interactions (S × K, Y × S × K) (Figure 2 and Figure 3). With the increasing potassium application rate, the SLRI and CS of each internode gradually increased. The SLRI and CS of each internode of the two straw treatments were the highest in the K120, while no significant difference between K90 and K120 was observed for the two indicators. With the increasing potassium application rate, compared with K0, the SLRI of K90 and K120 increased by an average of 83.3% and 95.8%, respectively, while the CS of the third to fifth internodes increased by an average of 64.3%, 62.9%, and 32.9%, respectively. Under the same potassium application rate, following the internodes shifting upward, the CS of three internodes showed that the third > fourth > fifth internode. Meanwhile, the CS of S1 was significantly higher by 19.8% than that of S0. The SLRI exhibited a similar trend, with the absolute value of S1 being 16.0% higher than that of S0 under the same potassium conditions.

### 2.3. Stem Morphological Characteristics

The internode dry weight (IDW), internode length (IL), and internode plumpness (IP) were significantly affected by the years (Y), straw (S), and potassium fertilizer (K) (the internode length of the fifth internode was not included). And their interactions (S × K, Y × S × K) had a highly significant effect on the IDW and IP in the third internode (Table 1). With the increasing potassium application rate, the IDW significantly increased, but the IL decreased, resulting in a significant increase in the IP. Compared with K0, with the increasing potassium application rate, the IDW and IP averages increased by 34.0% and 54.3%, respectively, and the IL average decreased by 12.1%. The most values of the IDW, IL, and IP were all at K120, while no significant difference between K90 and K120 was observed for them. The IP of S1 was significantly higher than S0 due to its higher IDW (on average 29.4% higher) and lower IL (on average 5.5% lower) under the same potassium application rate. In addition, as the internodes went upward, the IL gradually increased, while the IDW and IP showed a gradually decreasing trend, with the largest variation in the third internode. Among them, compared with S0, the IDW of the third, fourth, and fifth internodes under S1 treatment increased by averages of 44.2%, 9.1%, and 21.2%; the IL decreased by averages of 7.5%, 7.6%, and 0.4%; and the IP increased by 37.2%, 26.7%, and 24.5% on average, respectively.

### 2.4. Stem Soluble Sugar Content

**Figure 4:**
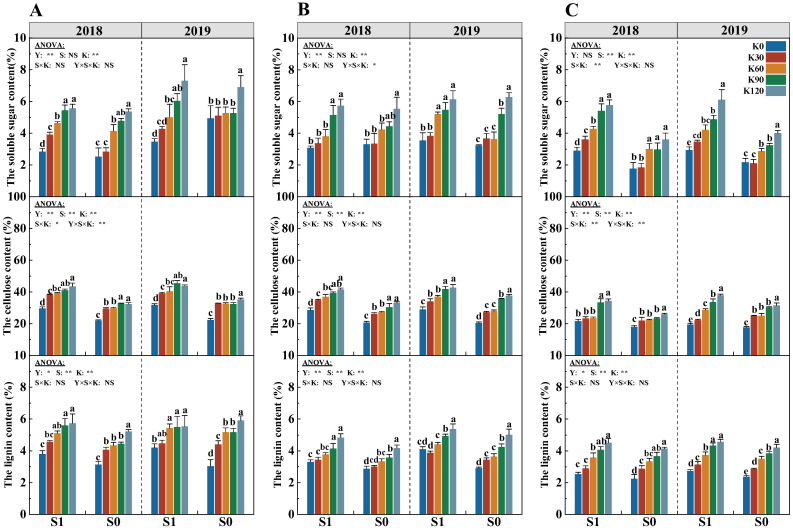
Effects of straw return and potassium fertilizer on the soluble sugar content, cellulose content, and lignin content of the third (**A**), fourth (**B**), and fifth (**C**) internodes. Y, S, and K represent year, straw treatment, and potassium fertilizer treatment, respectively. Values within a column followed by different lowercase letters indicate significant differences within treatments (*p* < 0.05). The error bar indicates the standard deviation (S.D., n = 3). * and ** indicate differences significant at the 0.05 and 0.01 levels, respectively. NS, no significance.

The soluble sugar content was significantly affected by potassium fertilizer (K) and straw (S), and their interactions (S × K) significantly affected the soluble sugar content of the fifth internode (Figure 4). With the increasing potassium application rate, the soluble sugar content of each internode significantly increased. Compared with K0, the soluble sugar content of the third, fourth, and fifth internodes averaged increases of 54.8%, 42.7%, and 56.2%, respectively. The soluble sugar content of each internode was the highest in the K120, while no significant difference between K90 and K120 was observed for each internode of S1. Under the same potassium application rate, the soluble sugar content of S1 was significantly higher by 23.6% than that of S0.

### 2.5. Stem Cellulose and Lignin Content

The cellulose and lignin content were significantly affected by the years (Y), straw (S), and potassium fertilizer (K), while their interactions (S × K, Y × S × K) only significantly affected the cellulose content of the third and fifth internodes (Figure 4). With the increasing potassium application rate, the cellulose and lignin content of each internode significantly increased. Compared with K0, the cellulose and lignin content increased by an average of 42.2% and 39.8%, respectively. The cellulose content increased the most in the third internode (30.8%), and lignin content increased the most in the fourth internode (17.3%). The cellulose and lignin content of two straw treatments were the highest in the K120, while no significant difference between K90 and K120 was observed for the cellulose and lignin content of S1. Under the same potassium application rate, the cellulose and lignin content of each internode gradually decreased as the internodes moved upwards. The cellulose and lignin content of S1 were significantly higher by 25.0% and 13.4% than that of S0, respectively.

### 2.6. Lignin Synthesis Enzyme Activity

**Figure 5:**
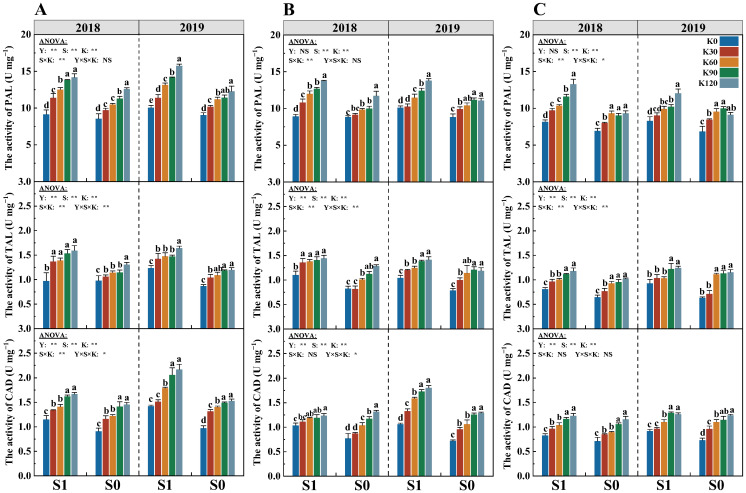
Effects of straw return and potassium fertilizer on the activity of phenylalanine ammonia lyase (PAL), tyrosine ammonia lyase (TAL), and cinnamyl alcohol dehydrogenase (CAD) of the third (**A**), fourth (**B**), and fifth (**C**) internodes. Y, S, and K represent year, straw treatment, and potassium fertilizer treatment, respectively. Values within a column followed by different lowercase letters indicate significant differences within treatments (*p* < 0.05). The error bar indicates the standard deviation (S.D., n = 3). * and ** indicate differences significant at the 0.05 and 0.01 levels, respectively. NS, no significance.

The enzyme activities of phenylalanine ammonia-lyase (PAL), tyrosine ammonia-lyase (TAL), and cinnamyl alcohol dehydrogenase (CAD) were significantly affected by the straw (S) and potassium fertilizer (K), while their interaction (S × K) only significantly affected the enzyme activities of PAL, TAL, and CAD of the third internode (Figure 5). With the increasing potassium application rate, the activities of PAL, TAL, and CAD of each internode significantly increased, and their activities of the two straw treatments were the highest in the K120. Compared with K0, the enzyme activities of PAL, TAL, and CAD increased by 29.1%, 33.8%, and 39.6%, respectively. Under the same potassium application rate, the activities of PAL, TAL, and CAD of S1 were significantly higher by 17.0%, 24.9%, and 21.4% than those of S0, respectively. Compared with the fourth and fifth internodes, the activities of PAL, TAL, and CAD in the third internode increased significantly, with an average increase of 23.6%.

**Figure 6:**
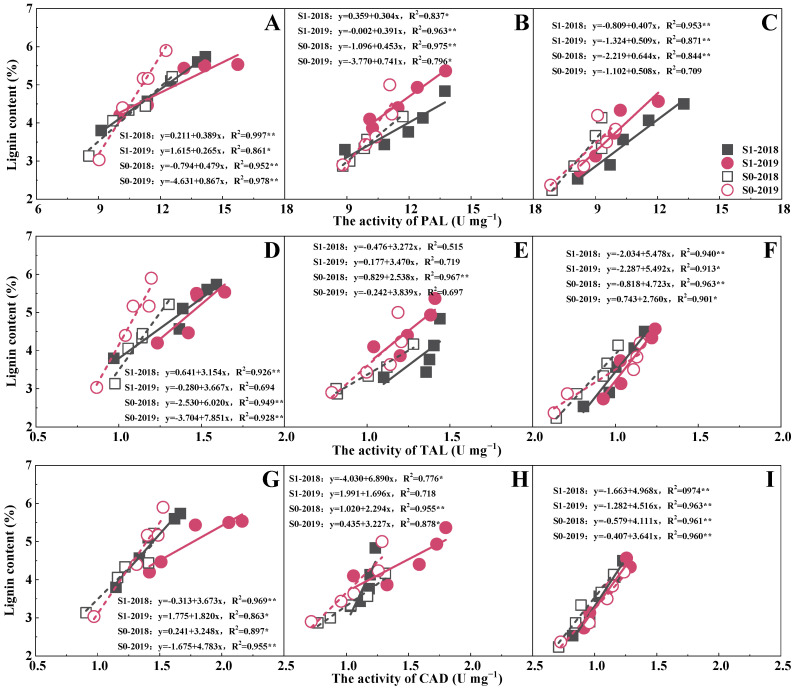
The correlation between lignin and phenylalanine ammonia lyase (PAL; (**A**–**C**)), tyrosine ammonia lyase (TAL; (**D**–**F**)), and cinnamyl alcohol dehydrogenase (CAD; (**G**–**I**)) enzyme activities of the third (**A**,**D**,**G**), fourth (**B**,**E**,**H**), fifth (**C**,**F**,**I**) internodes. Values within a column followed by different lowercase letters indicate significant differences within treatments (*p* < 0.05). * and ** indicate differences significant at the 0.05 and 0.01 levels, respectively. NS, no significance.

In different treatments and internodes, lignin content increased with the increase in enzyme activity (Figure 6). There was a significant positive correlation between lignin content and enzyme activity. In the third and fourth internodes, the correlation between PAL activity and lignin content was stronger (R^2^ was greater), while in the fifth internode, CAD had a stronger correlation. Compared with S0, the effect of PAL enzyme activity on lignin content was stronger in the S1 treatment and had the strongest correlation under the third internode (Figure 6A–C). The two-year results showed that under the same enzyme activity, the lignin content of S1 increased across two years, while the difference under S0 was not significant. For the TAL enzyme, the two-year results showed that in the third and fifth internodes, under the same enzyme activity, the lignin content increased across two years, with the enzyme activity showing a significant correlation (Figure 6D–F). But in the fourth internode, the correlation between lignin content and enzyme activity was not significant. For the CAD enzyme, compared with the third and fourth internodes, the fifth internode had the strongest correlation, but the difference between S1 and S0 was not significant in this internode (Figure 6G–I). In the third and fourth internodes, under the same enzyme activity, the lignin of the S1 treatment decreased across two years, while that of S0 increased year by year. However, compared with S0, S1 had higher CAD enzyme activity and lignin content.

### 2.7. Soil Potassium Balance and Available Potassium Content

**Figure 7:**
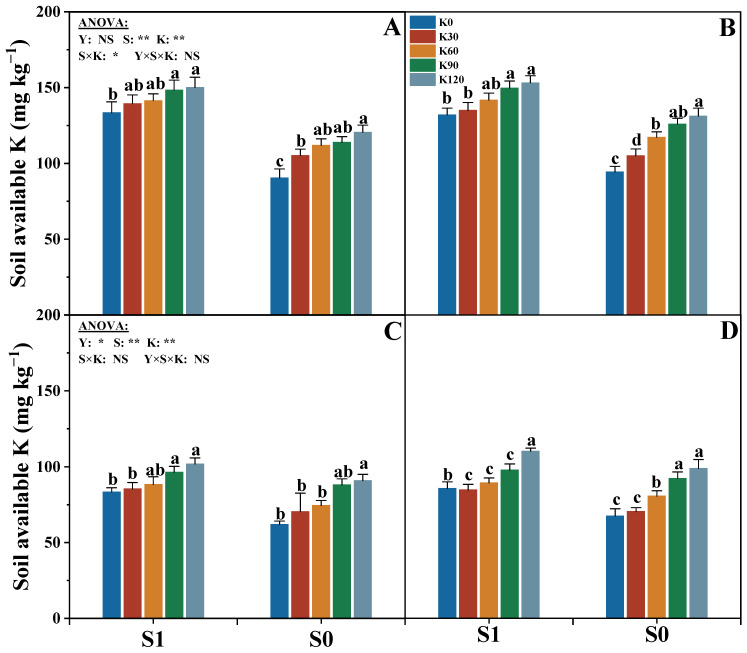
Effects of straw return and potassium fertilizer on soil available potassium content in the 0–20 cm (**A**,**B**) and 20–40 cm (**C**,**D**) soil layers in 2018 (**A**,**C**) and 2019 (**B**,**D**). Y, S, and K represent year, straw treatment, and potassium fertilizer treatment, respectively. Values within a column followed by different lowercase letters indicate significant differences within treatments (*p* < 0.05). The error bar indicates the standard deviation (S.D., n = 3). * and ** indicate differences significant at the 0.05 and 0.01 levels, respectively. NS, no significance.

The soil available potassium content in the 0–20 cm and 20–40 cm layers was significantly affected by the straw (S) and potassium fertilizer (K) (Figure 7). With the increasing potassium application rate, the soil available potassium content in the 0–20 cm and 20–40 cm layers of the two straw treatments increased significantly. Compared with K0, the available potassium content in the 0–20 cm and 20–40 cm soil layers averaged increases of 17.6% and 20.2%, respectively, and their content of the two straw treatments was the highest in the K120, while there was no significant difference between K90 and K120. Under the same potassium application rate, the soil available potassium content in the 0–20 cm and 20–40 cm layers of S1 were significantly higher by 21.9% and 14.2% than that of S0, respectively.

**Figure 8:**
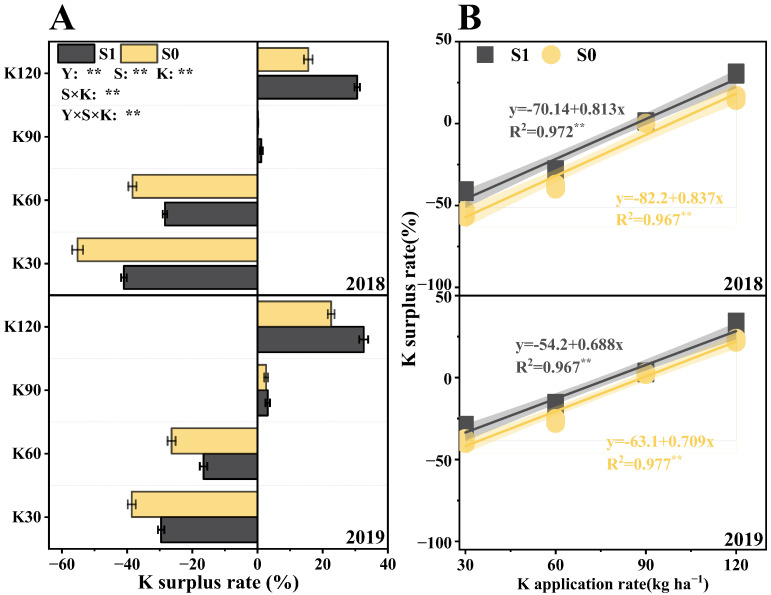
Effects of straw return and potassium fertilizer on K surplus rate (**A**) and the correlation of K surplus rate with K application rate (**B**). Y, S, and K represent year, straw treatment, and potassium fertilizer treatment, respectively. Values within a column followed by different lowercase letters indicate significant differences within treatments (*p* < 0.05). The error bar indicates the standard deviation (S.D., n = 3). * and ** indicate differences significant at the 0.05 and 0.01 levels, respectively. NS, no significance.

The K surplus rate was significantly affected by the years (Y), straw (S), potassium fertilizer (K), and their interactions (S × K, Y × S × K) (Figure 8A). The K surplus rate gradually increased with the increasing potassium application rate. Compared with S0, S1 presented 91% higher values in the K surplus rate across two years under the same potassium application rate. The soil potassium achieved a basic balance when the potassium application rate was 90 kg ha^−1^, while soil potassium showed a surplus state when the application rate was 120 kg ha^−1^. The fitting analysis of K surplus rate and K application rate indicated that when soil potassium of S1 was basically balanced across two years, the potassium fertilizer application rates were 86.2 kg ha^−1^ and 78.8 kg ha^−1^, respectively, while when soil potassium of S0 was basically balanced across two years, the potassium fertilizer application rates were 98.2 kg ha^−1^ and 89.0 kg ha^−1^, respectively (Figure 8B). Compared with the S0, the potassium input of S1 averaged a decrease of 11.8% across two years when soil potassium was basically balanced.

### 2.8. Correlation Analysis

**Figure 9:**
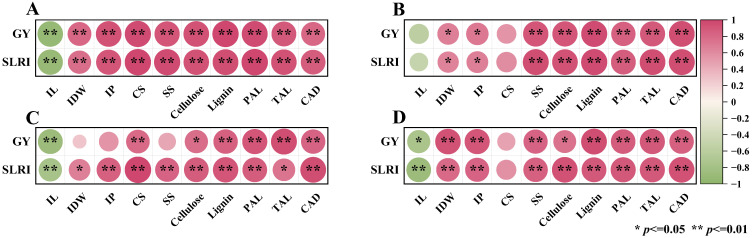
The correlation analysis of lodging resistance of the third and fifth internodes under different straw treatments. Note: (**A**) the third internode of S1 treatment, (**B**) the fifth internode of S1 treatment, (**C**) the third internode of S0 treatment, (**D**) the fifth internode of S0 treatment. GY, grain yield; IL, internode length; IDW, internode dry weight; IP, internode plumpness; SLRI, stem lodging resistance index; CS, crushing strength; SS, soluble sugar; PAL, phenylalanine ammonia-lyase activity; TAL, tyrosine ammonia-lyase activity; CAD, cinnamyl alcohol dehydrogenase activity. * and ** indicate differences significant at the 0.05 and 0.01 levels, respectively. NS, no significance.

The correlation analysis results suggested that the grain yield (GY) and stem lodging resistance index (SLRI) of the third internode of S1 treatment show an extremely significant positive correlation with internode dry weight (IDW), internode plumpness (IP), crush strength (CS), soluble sugar content, cellulose content, lignin content, and enzyme activities (PAL, TAL, and CAD), while the GY and SLRI of the third internode of S1 treatment were significantly negatively correlated with IL (Figure 9A). In contrast, in the third internode of the S0 treatment, GY was not significantly correlated with IDW, IP, and SS (Figure 9C). The fifth internode of S1 treatment showed an extremely significant positive correlation with soluble sugar content, cellulose content, lignin content, and enzyme activities (PAL, TAL, and CAD), and a significant correlation with IDW and IP, and there was no significant correlation with IL and CS (Figure 9B). At the same time, under the S0 treatment of the fifth internode, GY and SLRI were not significantly correlated with CS but were significantly correlated with other indicators (Figure 9D).

## 3. Discussion

### 3.1. Response of Maize Yield to Straw Return and Potassium Fertilizer

Lodging is the main factor affecting crop yield, and it is estimated that maize yield is reduced by approximately 35% annually both domestically and internationally due to lodging [37]. Regulating crop management measures, such as proper potassium fertilizer application to soil, can improve its photosynthesis effectively, promoting dry matter accumulation [38], and also can improve soil physical conditions and increase effective nutrients in the soil [39]. Furthermore, the increased input of K to soil increases the stem diameter and plumpness of each internode, improves the content of lignin and cellulose in the stem basal internode, increases the thickness of the parenchyma tissue and mechanical tissue, enhances stem mechanical strength, and reduces crop lodging index, which are consistent with the findings of our results [40]. In our experiment, potassium fertilizer significantly increased maize yield (Figure 1). Notably, there was no significant difference between the K90 and K120 in maize yield, and the maize yield of S0 showed a decreasing trend from year to year, which means that single potassium fertilizer or excessive potassium fertilizer application could not obtain a sustainable increase in yield.

Straw return could effectively enhance soil properties, increase available nutrients that could be absorbed directly by crops, and subsequently improve maize yield [41]. This result indicated that the yield under the straw return and potassium fertilizer was significantly higher than the no straw return treatment (single potassium fertilizer application) across two years. Compared with 2018, the grain yield increased by 6.9% in 2019 (Figure 1). Chen et al. [42] reported that straw return could reduce the impact of mineral fertilizer or NPK co-application on crop yield, which is consistent with our results. In this study, the maize yield of S0K90 was close to that of S1K60 in 2018, but in 2019, the maize yield of S1K60 was even significantly higher than that of S0K120 (Figure 1). This means that the negative impact of continuous potassium application on crop yield was reduced when straw was returned. In addition, straw return not only reduces the potassium fertilizer application rate, but also could achieve a more stable high yield.

### 3.2. Response of Stem Morphological Characteristics, Crushing Strength, and Lodging Resistance Index of Maize to Straw Return and Potassium Fertilizer

Lodging is mainly due to the imbalance between the aboveground and underground parts of crops, which is usually observed in the plant stems [43]. The crop resistance ability is closely related to the stem morphological structure and mechanical strength [11]. Specifically, shorter internodes of the stem (basal stem) correlate with greater mechanical strength and stem lodging resistance. The stem lodging resistance index (SLRI) significantly improved with increasing potassium application. Straw return combined with high potassium application could have a greater increase on the LRI compared with high potassium application without straw return in this study (Figure 2), and the higher LRI of straw return combined with potassium fertilizer application was attributed to its greater internode dry weight (IDW), internode plumpness (IP), stem crush strength (CS), and shorter internode length (IL) (Figure 3 and Table 1). This may be due to K input, which could influence the root development and enhance maize physiological metabolic activities, facilitate cell division and elongation, accelerate stem growth rate, thereby improving resistance to adversity [44], and the application of straw could enhance the K absorption in soil by maize, which is conducive to improving the stem strength [40]. Interestingly, the SLRI and related characteristics such as IDW, IP, and CS had the highest absolute value at K120 (the maximum potassium application rate); however, there were no significant differences between the K90 and K120 observed for these characteristics. This suggests that the appropriate potassium fertilizer application rate had a positive effect on improving stem lodging resistance, while excessive K input may negatively influence the increase in lodging resistance. Consequently, straw return combined with appropriate potassium fertilizer (e.g., 90 kg ha^−1^) significantly increased the CS by increasing IDW and IP and decreasing the IL, thereby improving the SLRI.

### 3.3. Response of Stem Physiological Characteristics and Lodging Resistance Index of Maize to Straw Return and Potassium Fertilizer

In comparison to the stem structural characteristics of plants, soluble sugar, cellulose, lignin, and other chemical components serve as the internal physiological foundation for the stem structure and strength and have a relatively direct impact on enhancing the thickness and toughness of the stem wall, improving the stem mechanical strength, and improving the crop lodging resistance [45]. Soluble sugar is the non-structural carbohydrate stored in the stem, the amount of which represents the resistance of the stem to the external adverse environment. A deficiency in soluble sugar can inhibit stem growth and reduce the internode strength [46]. Previous research has demonstrated that an increase in the accumulation of soluble sugars could enhance stem internode plumpness (IP), thereby enhancing stem lodging resistance [47]. Our results indicated that there was a strong significant correlation between the soluble sugar content and SLRI (Figure 9), and straw return and potassium fertilizer application both could significantly increase the soluble sugar content, and the combination of the two was more effective (Figure 3). The potassium fertilizer application could increase the soluble sugar accumulation in the stems in the form of non-structural carbohydrates, which contributed to maintaining the upright strength of the stem [48]. Simultaneously, this positive effect was enhanced by straw application, which involved more potassium in the physiological and biochemical reactions within the plant [49].

Cellulose and lignin were the main components of the plant cell wall, which directly affect the stem mechanical strength and crop lodging resistance [50,51]. PAL, TAL, and CAD are critical enzymes in the lignin synthesis process of plants, which play an important role in the resistance response of plants [10]. Previous studies found that insufficient lignin in the stems, lower cellulose content, and related enzyme activities all lead to crop lodging [49,52,53]. The potassium fertilizer application could significantly increase lignin-related gene expression abundance and enzyme activity, induce lignin biosynthesis, and enhance the stem mechanical strength and crop lodging resistance [54]. Furthermore, Li et al. [55] found that the interaction of fertilization practices and straw return could maintain a better nutrient supply, and the adequate nutrient supply could maintain lignin synthesis-related enzyme activities in stems at high levels [56]. This study showed that straw return combined with potassium fertilizer significantly increased lignin, cellulose, and the activities of PAL, TAL, and CAD compared to the single potassium fertilizer input or straw (Figure 3 and Figure 4). The result may be due to the fact that potassium application could directly increase the supply of potassium in the stems, which promotes lignin synthesis by increasing enzyme activity. In addition to NPK, Ca, Mg, S, and organic matter are also abundant in straw [57]. Ca plays an important role in determining the structure and function of the cell wall. Ca combines with pectin acid to form pectin–calcium, fixed in the intercellular layer, so that adjacent cells adhere to each other so as to maintain the stability of cell structure and normal function [58], and the participation of Mg can affect the changes in soluble sugar, cellulose, and lignin [59]. Studies have shown that the participation of exogenous Ca^2+^ could up-regulate the transcription level of PAL-related genes [60], and K^+^ from straw and exogenous application could ensure the smooth progress of metabolic reactions, and K^+^ could maintain cell turgor and elongation, providing greater surface area and space for lignin deposition.

Stem lodging can occur in all basal internodes of maize, with approximately 75% of stem lodging occurring between the third and fifth internodes [61]. In this study, we analyzed the correlation between the morphological characteristics, crushing strength, and physiological characteristics of the third and fifth internodes with grain yield and stem lodging resistance index (Figure 9). The results showed that compared with the fifth internode, the crushing strength of the third internode had a more significant correlation with grain yield and stem lodging resistance index. At the same time, compared with S0, the correlation between the morphological and physiological characteristics of the third internode with the stem lodging resistance index was more significant under S1 treatment. For the fifth internode, the application of no straw increased the correlation coefficient between the morphological characteristics of the fifth internode with grain yield and lodging resistance index. This may mean that straw returning combined with potassium fertilizer could significantly enhance the mechanical strength of the third internode by improving the physiological and morphological characteristics of the third internode, thereby improving the lodging resistance of the stem. It was found that straw return could significantly increase the content of cellulose and lignin in the third internode and related synthase activities, significantly enhance the mechanical strength of the third internode, and thus significantly improve the stem lodging resistance [62], which was consistent with findings in this study.

### 3.4. Response of Soil Nutrient Balance to Straw Return and Potassium Fertilizer

Great soil productivity is the basis for high crop yields. Soil potassium content is an important source of K absorption for crops; however, the available potassium in soil (including water-soluble potassium (WSK), exchangeable potassium (EK), and non-exchangeable potassium (NEK)) accounted for only 0.1% to 10% of total soil potassium [63,64]. Increasing K application is an effective strategy for enhancing the available K content in farmland, and the recycling and utilization of straw could facilitate potassium absorption for crops by enhancing soil structure, thereby resulting in higher available potassium content and supply capacity [65]. It was found that straw return combined with potassium application could increase EK and NEK content and storage, and potassium application could promote an increase in soil potassium supply by expanding the EK capacity in soil macroaggregates, and straw return could significantly improve the abundance of soil macroaggregates and EK/NEK capacity [66]. Our study indicates that both potassium application and straw return could increase the soil available potassium content (Figure 7). This may be due to a direct increase in soil potassium content by the input of exogenous fertilizer potassium and straw potassium. The appropriate potassium fertilizer application could improve crop yields and increase nutrient uptake while improving the soil potassium supply capacity [66]. Compared with K0, although the increase in available potassium content of the S0 was significantly higher than that of the S1, the available potassium content in different soil layers of the S1 was markedly higher than that of the S0 (Figure 7).

The K surplus rate in soil depended on the absolute value of soil potassium input and output, which can directly reflect the dynamic fluctuations of potassium inputs and consumption. Sufficient potassium was not provided by weathering of mineral potassium to support plant growth in crop rotation, so the main sources of nutrients for plant growth were inherent in the soil and exogenous potassium inputs. Potassium fertilizer and straw return were important methods for nutrient supplementation. Straw return not only could alleviate soil potassium deficiency [67] but also improve soil quality by increasing the proportion of macroaggregates and soil organic carbon (SOC) content [68]. In our study, soil potassium achieved a basic balance when the potassium application rate was 90 kg ha^−1^, while soil potassium showed a surplus state when the application rate was 120 kg ha^−1^ (Figure 8A). Moreover, at the same potassium application rate, the S1 showed a higher surplus rate, which indicated that the soil potassium reached dynamic equilibrium more quickly under straw return combined with potassium fertilizer conditions.

It was not enough to meet the needs of high and stable crop yields and maintain the soil potassium balance by only relying on straw return, and excessive K input could lead to resource waste. Our study demonstrated that straw return combined with potassium fertilizer could enhance the soil’s available potassium content by 18.1% (Figure 7), compared with a single potassium fertilizer application. And the fitting results showed (Figure 8B) that, in comparison to single potassium fertilizer application, the potassium application rate that achieved the dynamic equilibrium of soil potassium balance in two years was reduced by about 11.8%, although the highest values of soil nutrients and yield were obtained at K120, while there was no significant difference between K90 and K120 for the soil nutrient and yield, and it was more favorable to maintain soil nutrient balance under K90. Therefore, the results of this study showed that straw return combined with potassium fertilizer is more effective than a single potassium fertilizer application. The combination of straw return and potassium fertilizer application could not only reduce soil potassium deficiency caused by crop absorption but also enhance soil potassium cycling and supply capacity [69], and this means that the aboveground part of the crop is able to absorb more potassium from soil under the condition of straw return combined with potassium fertilizer, which is used to supply the crop growth and provide support for improving the crop lodging resistance.

## 4. Materials and Methods

### 4.1. Experimental Site

This study was conducted in 2018 and 2019 based on a long-term field trial with straw return combined with chemical fertilizer since 2014 at the Western Experimental Station of Jilin Agricultural University (45°26′ N, 124°87′ E). The test area has a mesothermal continental monsoon climate, with an average annual sunshine duration of 2643.3 h, annual precipitation of 599.4 mm, and a frost-free period of 139 days. The test soil is chernozem. The initial characteristics of the soil at 20 cm depth before the experiment were determined as follows: organic matter content was 14.7 g/kg, total N content was 2.13 g/kg, total P content was 0.53 g/kg, total K content was 18.1 g/kg, available N content was 75.91 mg/kg, available P content was 16.31 mg/kg, available K content was 130.24 mg/kg, and pH value was 7.24.

### 4.2. Experimental Design

In this experiment, a wide-narrow row planting pattern was adopted, with narrow rows of 40 cm, wide rows of 90 cm, and a row height of 12 cm. A two-factor split-plot design was used, with straw return (S1) and no straw return (S0) set as the main factor plot. Hengdan188, a maize cultivar, was sown at 75,000 plants ha^−1^. After the maize harvest, the straw was directly removed from the no-straw return area (S0) since 2014, while in the straw return area (S1), the straw was cut short (<10 cm in length) with a chopper and evenly dispersed over the field, and the straw was turned deep into 30–40 cm of soil with a turning plow and then rotated and raked by a hydraulic offset heavy harrow machine and a combined ground preparation machine to achieve the sowing state. The amount of straw return was 9000 kg ha^−1^ (dry weight), and the K content was 90 kg ha^−1^, and the pre-crop was maize.

The potassium fertilizer was the subplot, which included five K fertilizer (K_2_O) levels: 0 (K0), 30 (K30), 60 (K60), 90 (K90), and 120 (K120) kg ha^−1^. All treatment areas were applied with 100 kg ha^−1^ P (P_2_O_5_), and a total of 200 kg ha^−1^ N (Urea) fertilizer with a ratio of 3:7 was applied before maize sowing as a base fertilizer and during the anthesis stage (VT), respectively. Both P and K fertilizers were applied at once before sowing. Each plot was 14.5 m × 20.8 m (302 m^2^) and was replicated three times. Maize was sown with mechanization on 28 April and 29 April over two consecutive years (2018–2019) and harvested on 3 October in both years.

### 4.3. Data Collection

#### 4.3.1. Stem Lodging Resistance Indicators and Morphological Characteristics

At the silking stage (R1), 10 uniform plants were selected from each plot. All plant parts attached to the main stem were removed, such as ears and leaf sheaths. The main stem was divided according to different internodes, and the basal third to fifth internodes were selected to measure each internode length (IL, cm) and fresh weight.

After the measurement of fresh weight, a plant lodging tester (TP-YDD-1, Hangzhou, China) was used to evaluate the mechanical strength of each sample’s internodes. The internode to be tested was placed on the instrument’s testing platform, and a probe with a cross-sectional area of 1 cm^2^ was slowly pressed vertically onto the stem until it broke, reading the maximum value (N), which is defined as the crush strength (CS). The lodging resistance index (LRI) was calculated as:

(1) LRI=CS of the 3rd internode/CGH

After the determination of stem crush strength (CS), the test fresh samples were divided into two parts. One part was heated at a constant temperature of 105 °C for 30 min, followed by drying at 80 °C until a constant weight was achieved to obtain the internode dry weight (IDW, g) and calculate the internode plumpness (IP, mg/cm), and the other part of the test fresh sample was used for the subsequent determination of the stem physicochemical properties. The internode plumpness (IP, mg/cm) was calculated as:

(2) IP= IDW/IL

#### 4.3.2. Stem Soluble Sugar, Cellulose, Lignin Content, and Enzyme Activity

(i) The dry samples from Section 4.3.1 were ground into powder and sieved (100 mesh). The obtained sample powder is placed in a drying bag and stored at 4 °C for the determination of lignin and other related indicators.

The lignin content is determined using the Cysteine-Assisted Sulfuric Acid (CASA) method [70]. Weigh 5 mg of the powder into a test tube, add 1 mL of 72% sulfuric acid containing 0.1 g/mL cysteine, stir, and react at room temperature (24 °C) for 1 h. After the reaction, dilute the solution to 100 mL, and then measure the absorbance of the solution at 283 nm UV. The lignin content can be calculated from this measurement. The cellulose content was measured using a fiber analysis system following the methods of Van Soest et al. (1991) [71]. The soluble sugar content was measured by the anthrone colorimetric method [72].

(ii) The fresh samples for stem crush strength determination in 4.3.1 were used to analyze the enzyme activity in stems. The phenylalanine ammonia-lyase (PAL) and tyrosine ammonia-lyase (TAL) are measured using the method of Gao et al. [73], which is an improvement based on the method of Kováčik et al. [74].

The cinnamyl alcohol dehydrogenase (CAD) activity is determined with reference to the method of Goffner et al. [75], with slight modifications. Grind 1.5 g of maize stem sample in liquid nitrogen, add 1 mL of pre-cooled 0.1 mmol/L pH 6.25 PBS (containing 15 mmol/L mercaptoethanol, 2% PEG, and about 0.1 g PVPP), mix thoroughly by vortexing, and then centrifuge at 4 °C at 18,000 r/min for 20 min. The supernatant is the enzyme extract. Using trans-cinnamaldehyde as the substrate, one enzyme activity unit (U) is defined as a change of 0.01 in OD340nm per hour, and the specific enzyme activity (U/mg) is calculated.

#### 4.3.3. Soil Available K Content

After the maize harvest, soil samples were collected from each treatment area in the experimental year. In each area, five points were randomly selected to sample 0–20 and 20–40 cm of soil, after which samples of the same depth were mixed. The available K in the soil was extracted using the method of Knudsen et al. [76] to determine its content.

#### 4.3.4. Soil K Surplus Rate

Sampling at the maize harvest. The maize plants were separated into various organs, then heated at a constant temperature of 105 °C for 30 min, and dried at 80 °C until a constant weight was attained. After determining the dry weight of each organ, they were ground into powder. The K content was determined by the flame photometry method, following the approach of Jones and Case [77]. The K surplus rate was calculated as:

(3) $$K\;\mathrm{surplus}\;\mathrm{rate}=\frac{(\mathrm{Total}\;K\;\mathrm{input}-\mathrm{Total}\;K\;\mathrm{absorbed}\;\mathrm{by}\;\mathrm{the}\;\mathrm{crop})}{\;\mathrm{Total}\;K\;\mathrm{absorbed}\;\mathrm{by}\;\mathrm{the}\;\mathrm{crop}}\times 100\%$$

The total K input mainly refers to the input of chemical fertilizer and organic fertilizer, and the total K output mainly includes the amount of crop aboveground. Rainfall, potassium brought in by seeds, and potassium taken away by surface runoff were not included.

#### 4.3.5. Grain Yield

At the maize maturity stage, plants were harvested from an area of 26 m^2^ (4 rows, 10 m long) that was selected in the center of each treatment plot. In each treatment, 20 ears were randomly selected from the harvested ears based on the average panicle weight to determine grain yield (grain yields were standardized to 14% moisture).

### 4.4. Data Analysis

Data in this study were collated by Excel 2019, and Origin 2024 software was used for mapping. SPSS 26.0 software was used for multivariate analysis of variance, and the least significant difference method (LSD) at the probability level of 0.05 was used for multiple comparisons between treatments.

## 5. Conclusions

Both straw return and high potassium (K) fertilizer inputs could increase maize yield. However, the best results are achieved when straw return is combined with an appropriate K input of 90 kg ha^−1^, which not only enhances maize yield but also strengthens lodging resistance and improves soil nutrient balance. This synergistic effect is primarily due to the combined impact of straw return and K fertilization in improving stem lodging resistance and soil nutrient availability. These improvements are associated with increased crush strength, internode dry weight, internode plumpness, carbohydrate content, enzyme activities, and reduced basal internode length. Moreover, enhancing stem crush strength, especially at the basal third internode, plays a key role in improving lodging resistance. In conclusion, straw return combined with 90 kg ha^−1^ K fertilizer application could obtain the opportunity to increase yield by improving the stem lodging resistance and maintaining soil K balance. This study provides certain insights into the sustainable utilization of straw resources and the efficient utilization of chemical fertilizers in the spring maize planting in Northeast China, and aims to achieve high and stable maize yield while enhancing maize resistance.

## Figures and Tables

**Table 1:** Effects of straw return and potassium fertilizer on the morphological characteristics of the third, fourth, and fifth internodes.

Year	S	K	Internode Dry Weight (g)			Internode Length (cm)			Internode Plumpness (mg/cm)		
	Treatment	Treatment	3rd	4th	5th	3rd	4th	5th	3rd	4th	5th
2018	S1	K0	4.5 c	5.4 d	4.8 b	15.1 a	17.2 a	19.4 c	300.1 d	315.7 c	250.7 b
		K30	6.3 b	7.1 bc	6.6 a	13.7 b	14.7 b	20.6 ab	459.5 c	480.1 b	320.9 b
		K60	6.6 b	6.5 c	6.1 a	12.9 bc	14.2 b	20.7 a	508.9 b	456.2 b	297.2 b
		K90	7.9 a	7.9 ab	6.6 a	12.2 cd	12.6 c	19.7 c	650.0 a	624.2 a	337.1 ab
		K120	7.9 a	8.4 a	6.8 a	11.4 d	12.1 c	19.8 bc	689.5 a	697.1 a	344.6 a
	S0	K0	3.5 d	5.2 b	4.1 c	15.9 a	18.1 a	22.7 a	217.4 d	293.5 b	181.8 c
		K30	4.4 c	5.5 b	5.3 b	14.4 b	17.1 a	20.5 b	308.1 c	322.1 b	260.9 b
		K60	5.5 b	6.3 ab	5.8 ab	13.8 c	14.7 b	19.2 c	398.4 b	428.6 ab	301.5 ab
		K90	6.2 a	6.7 ab	6.0 ab	13.2 d	12.7 c	19.0 c	472.1 a	530.9 a	317.1 a
		K120	6.4 a	7.4 a	6.4 a	13.6 cd	13.2 c	18.7 c	468.5 a	563.1 a	342.2 a
2019	S1	K0	6.7 d	5.3 c	6.4 b	14.9 a	15.2 a	20.7 a	452.2 c	348.7 c	307.1 c
		K30	7.8 c	5.8 bc	6.9 b	14.3 ab	13.8 ab	20.4 a	546.6 b	421.4 bc	340.2 bc
		K60	9.1 a	6.2 b	8.0 a	13.4 bc	13.9 ab	18.3 c	679.5 a	448.8 b	434.9 a
		K90	8.7 ab	7.8 a	7.1 b	12.4 c	14.3 ab	18.5 bc	698.1 a	548.3 a	381.9 b
		K120	8.0 bc	7.7 a	7.0 b	12.4 c	13.3 b	19.2 b	648.5 a	583.4 a	364.0 b
	S0	K0	6.3 a	4.2 c	4.2 b	16.3 a	17.0 a	21.6 a	389.0 a	247.1 c	194.3 c
		K30	6.8 a	5.0 b	5.2 a	14.7 b	15.9 b	19.2 b	460.0 a	316.9 b	272.0 b
		K60	7.0 a	5.6 b	5.6 a	14.9 b	16.1 b	20.3 ab	471.0 a	344.4 b	276.3 b
		K90	6.2 a	6.6 a	5.9a	13.6 c	15.2 c	18.7 b	453.5 a	435.2 a	317.2 a
		K120	6.1 a	6.9 a	5.8 a	13.0 c	15.1 c	18.7 b	466.2 a	454.9 a	308.4 a
ANOVA											
Y			**	**	**	*	*	**	**	**	**
S			**	**	**	**	**	NS	**	**	**
K			**	**	**	**	**	**	**	**	**
S × K			**	NS	NS	NS	*	**	**	NS	*
Y × S × K			*	NS	NS	NS	NS	**	*	NS	**

Note: Y, S, and K represent year, straw treatment, and potassium fertilizer treatment, respectively. Values within a column followed by different lowercase letters indicate significant differences within treatments (*p* < 0.05). * and ** indicate differences significant at 0.05 and 0.01 levels, respectively. NS, no significance.

## Data Availability

Data is contained within the article.
